# Pediatric Inflammatory Multisystem Syndrome and Rheumatic Diseases During SARS-CoV-2 Pandemic

**DOI:** 10.3389/fped.2020.605807

**Published:** 2020-12-04

**Authors:** Adrien Schvartz, Alexandre Belot, Isabelle Kone-Paut

**Affiliations:** ^1^Service De Rhumatologie Pédiatrique, Centre De Référence Des Maladies Auto-Inflammatoires et de l'Amylose Inflammatoire, Hospital Bicêtre, Assistance Publique des Hôpitaux de Paris, Université Paris Sud Saclay, Le Kremlin-Bicêtre, France; ^2^Service de Néphrologie, Rhumatologie, Dermatologie Pédiatriques, Centre de Référence des Rhumatismes Inflammatoires et Maladies Auto-Immunes Rares de l'Enfant (RAISE), Hôpital Femme-Mère-Enfant, Hospices Civils de Lyon, Bron, France

**Keywords:** COVID - 19, pediatric inflammatory multisystem syndrome, SARS – CoV – 2, children, pediatric rheumatic disease, Multisystem Inflammatory Syndrome in Children (MIS-C)

## Abstract

Globally, the coronavirus disease 2019 (COVID-19), caused by severe acute respiratory syndrome coronavirus 2 (SARS-CoV-2), appeared to have a milder clinical course in children compared to adults. As severe forms of COVID-19 in adults included an aberrant systemic immune response, children with chronic systemic inflammatory diseases were cautiously followed. No evidence for a specific susceptibility was identified in this pediatric population. European and US Pediatricians started to notice cases of myocarditis, sharing some features with toxic shock syndrome, Kawasaki disease, and macrophage activation syndrome in otherwise healthy patients. Multisystem Inflammatory Syndrome in Children (MIS-C) and Pediatric Inflammatory Multisystem Syndrome (PIMS) have designated this new entity in the US and Europe, respectively. The spectrum of severity ranged from standard hospitalization to pediatric intensive care unit management. Most patients had a clinical history of exposure to COVID-19 patients and/or SARS-COV2 biological diagnosis. Clinical presentations include fever, cardiac involvement, gastro-intestinal symptoms, mucocutaneous manifestations, hematological features, or other organ dysfunctions. The temporal association between the pandemic peaks and outbreaks of PIMS seems to be in favor of a post-infectious, immune-mediated mechanism. Thus, SARS-CoV2 can rarely be associated with severe systemic inflammatory manifestations in previously healthy children differently from adults highlighting the specific need for COVID-19 research in the pediatric population.

## Introduction

Severe Acute Respiratory Syndrome Coronavirus type 2 (SARS-CoV-2) responsible for the Coronavirus disease 19 (COVID-19) pandemic has affected almost 50 million people leading to more than 1 million deaths around the world (1).

Originally, children were thought of having milder disease compared to adults (2–5). Besides, they were less affected overall with a percentage of 1.5% of cases occurring under 18 years old in early reports (5, 6). Recently updated national statistics in Canada, China, and Europe showed that, as of now, pediatric cases represent 2.1 to 8.4% of total confirmed COVID19 cases (7–9). Unlike adults, children did not develop a severe respiratory illness such as acute respiratory distress syndrome.

As reports underlined the magnitude of a cytokine storm in adults, patients receiving immunomodulatory treatment for either auto-inflammatory diseases, or autoimmune diseases received peculiar attention. Finally, until now, there have been no reports of concern for these patients and they did not show a higher risk for developing either COVID19 or its severe forms (10–12).

Against all odds, cases of hyperinflammatory syndromes emerged in otherwise healthy children. The first report came from intensive care physicians from the United Kingdom (UK). They described eight children aged from 4 to 14 years old with acute cardiac failure due to myocarditis in a general context of unrelenting fever, variable skin rash, conjunctivitis, distal edema, and generalized pain with substantial gastrointestinal symptoms. Even though all children tested negative for SARS-CoV-2 in bronchoalveolar wash or nasopharyngeal aspirates, they displayed important biological inflammatory markers and temporality with the COVID-19 pandemic, suggesting an association with SARS-CoV-2 (13).

The remarkable hyperinflammatory state of patients, as well as cardiac involvement, have initially, and no doubt improperly, brought this new disease to toxic shock syndrome, macrophage activation syndrome, and Kawasaki disease.

Since then, additional descriptive studies have offered a wider range of knowledge on the clinical heterogeneity of this new syndrome, now referred to as Pediatric Inflammatory Multisystem Syndrome (PIMS) or Multi Inflammatory Syndrome in Children (MIS-C) (14–38). Herein, we will use the term Pediatric Inflammatory Multisystem Syndrome (PIMS). In addition, these forms with late revelation have boosted the scientific interest already greatly accelerated by the COVID-19 pandemic, trying to better understand how and why the human immune system can react in various ways to the entry of a pathogen. We will describe the clinical presentation of patients with PIMS, with a highlight on cardiac dysfunction, and discuss the different treatment regimen they received. We will also discuss different pathophysiological hypotheses for PIMS and, finally, the specificity of patients with rheumatological diseases.

## Clinical Presentation of PIMS

### Main Symptoms

PIMS symptoms are diverse and non-specific. They commonly include persistent fever, mucocutaneous involvement (hands and feet oedema, conjunctivitis, swollen and cracked red lips, rash), cardiac dysfunction (myocarditis, electric abnormalities, valvular dysfunction, shock, coronary aneurysms or dilatation), gastrointestinal symptoms, and lymphadenopathy (13, 14, 16, 17, 19, 20, 28, 33, 34, 39).

Since the first alert by the UK and initial descriptions around the world, different clinical classifications have been proposed by the World Health Organization, the UK Royal College of Pediatrics and Child Health, the French “COVID pediatric inflammation” consortium, and the US Centre for Disease Control and Prevention (Appendix 1) (15, 40–42). Heart involvement was at the forefront in early descriptions. Cardiac shock was present in all of the eight children in the initial description (13) and was present in 80% of the 35 patients described by Belhadjer et al. (19). As more cases are reported, and inclusion criteria vary, heart involvement is no longer so predominant but remains a risk. In the study by Feldstein et al. with 186 patients reported, the two main organs involved were the gastrointestinal system in 171 patients (92%) and the cardiovascular in 149 (80%). The three other principal organs involved were the hematological system in 142 (76%) patients, 137 (74%) patients had mucocutaneous symptoms, and finally, 131 (70%) patients had respiratory symptoms (43). Verdoni et al. described 10 patients with 6 of them having diarrhea and five having hypotension (20). In the UK, Whittaker and colleagues reported abdominal pain in 31/58 (53%) patients, diarrhea in 30 (52%), and vomiting in 26/58 (45%). Mucocutaneous involvement was found in 30/58 (52%) patients with rash, 26/58 (45%) with conjunctivitis, and 17/58 (29%) with mucous membrane changes (14).

Initially described as a life-threatening condition, more recent reports suggest a wider clinical spectrum. In a US epidemiological study report, 570 PIMS patients who met the CDC case definition for PIMS (or MIS-C) were included. A total of 203/570 (35.6%) of them had a clinical course consistent with early published PIMS reports, characterized predominantly by shock, cardiac dysfunction, and abdominal pain. The remaining 367 patients had milder manifestations that appeared to overlap with acute COVID-19 or shared characteristics of KD (44).

We discuss 7 studies with clinical data for patients with PIMS (see Table 1). We included studies with at least 10 patients described but did not include the studies by Feldstein and Dufort as they are US studies with US patients reported in the epidemiological study we just discussed. This table features clinical data for 733 patients. Some data are missing due to differences in definitions or report by the study.

**Table 1:** Clinical features of PIMS patients.

**Study**	**Godfred-clato (44)**	**Whittaker (14)**	**Toubiana (45)**	**Pouletty (16)**	**Verdoni (20)**	**Belhadjer (19)**	**Webb (46)**
Number of patients	570	58	21	16	10	35	23
Age, median, (interquartile)	8 (4-12)	9 (5.7–14)	7.9 (3.7–16.6)	10 (4.7–12.5)	7.3 (5.4–8.5)	10 (NA)	6.59 (4.75–8.43)
Male	316 (55.4)	25 (43)	9 (43)	8 (50)	7 (70)	18 (51)	17 (73.9)
Ethnicity							
Hispanic	187 (40.5)						
Black, non–hispanic	153 (33.1)	22 (38)	24 (57)				18 (73.9)
White, non–hispanic	61 (13.2)	12 (21)	12 (29)				0
Other	30 (5.6)	6 (10)	2 (5)				5 (21.7)
Multiple	18 (3.9)						
Asian	13 (2.8)	18 (31)	4 (10)				
Unknown	108 (—)						
Organ involvement							
Gastrointestinal	518 (90.9)		21 (100)	13 (81)	6 (60)	29 (83)	
Abdominal pain	353 (61.9)	31 (53)					15 (65.2)
Vomiting	352 (61.8)	26 (45)					
Diarrhea	303 (53.2)	30 (52)			6 (60)		13 (56.5)
Cardiovascular	493 (86.5)						21 (91.3)
Shock	202 (35.4)	29 (50)		11 (69)	5 (50)	28 (80)	
Elevated troponin	176 (30.9)		17 (81)			35 (100)	
Elevated BNP^a^ or proBNP	246 (43.2)	29/29	14/18		10 (100)	28/28	
Congestive heart failure	40 (7.0)					35 (100)	8 (34.8)
Cardiac dysfunction	207 (40.6)					1 (3)	
Myocarditis	130 (22.8)		16 (76)	7 (44)			
Coronary artery dilatation or aneurysm	95 (18.6)	8 (14)	5 (24)	3 (19)	2 (20)	6 (17%)	1 (4.3)
Hypotension	282 (49.5)						13 (56.5)
Pericardial effusion	122 (23.9)		10 (48)	4 (25)	4 (40)		7 (30.4)
Mitral regurgitation	130 (25.5)				4 (40)		7 (30.4)
Dermatologic and mucocutaneous	404 (70.9)						
Rash	315 (55.3)	30 (52)	16 (76)	13 (81)	6 (60)	20 (57)	20 (87.0)
Mucocutaneous lesions	201 (35.3)	17 (29)	16 (76)	14 (87)			
Conjunctival injection	276 (48.4)	26 (45)	17 (81)	15 (94)	7 (70)		15 (65.2)
Swollen hands and feet		9 (16)		11 (68)	5 (50)		
Respiratory	359 (63.0)	12 (21)		2 (12)		23 (65)	10 (43.5)
Cough	163 (28.6)						
Shortness of breath	149 (26.1)						
Chest pain or tightness	66 (11.6)					6 (17)	
Pneumonia	110 (19.3)						
ARDS^b^	34 (6.0)						
Pleural effusion	86 (15.8)						
Neurologic	218 (38.2)		6 (29)	9 (56)		11 (31)	5 (22.7)
Headache	186 (32.6)	15 (26)					5 (22.7)
Confusion		5 (9)					
Meningism						11 (31)	
Renal	105 (18.4)						10 (43.5)
Acute kidney injury	105 (18.4)	13 (22)	11 (52)				
Other							
Periorbital oedema	27 (4.7)						
Lymphadenopathy	76 (13.3)	9 (16)	12 (57)			21 (60)	
Sore throat		6 (10)					
Anosmia				1 (6)			
Arthritis				1 (6)			8 (34.8)
Rhinorrhoea						15 (43)	

*Review of 7 studies with at least 10 patients reported. Total of patients: 733*.

a *B-type Natriuretic Peptide*.

b *Acute Respiratory Distress Syndrome. Gray cells are data for which the study does not give information. Every cell is displayed with the number of patients and (percentage). Cells with no percentages are when not all the patients of the study had reported data. Organ involvement are not detailed due to differences of definition by each study, it was included when organ affection was described. Gray cells are for when data were not available. Blue and orange cells are for presentation*.

As for other hyperinflammatory states, there are no specific diagnostic tests and diagnosis is made by an array of clinical signs and biological results.

#### Cardiac Dysfunction

Cardiac dysfunction appears the hallmark describing PIMS patients and includes coronary artery dilatation or aneurysms, myocarditis, left ventricular dysfunction, pericardial effusion, and even shock. Most of them were admitted to the PICU and required inotrope or vasoactive support (13, 19, 45, 46).

In their alert, Riphagen et al. described 8 patients, all requiring inotropic drugs (13). As they were recruited in the PICU, there is a bias but the patients of Belhadjer and colleagues had ejection fraction below 30% in 10/35 of patients, and between 30 and 50% in 25/35 patients (19). In their study of 21 patients, Toubiana et al. reported pericardial effusions occurring in almost half of their patients [10/21 (48%)] and pleural effusion in three (14%). Sixteen (76%) patients had myocarditis with a left ventricular ejection dysfunction under 60%. Out of these 16 patients, two displayed electric changes with an increased QT interval or diffuse ST-segment elevation.

Cardiovascular involvement is particularly important in these patients and regular evaluations with an electrocardiogram (ECG) and echocardiogram are necessary. The monitoring of cardiac enzymes is of great interest. Elevation of troponin, B-type natriuretic peptide (BNP), pro-B-type natriuretic peptide (proBNP) levels suggest myocardial damage and heart failure. In their study with 33 patients, Kaushik et al. reported elevated median levels of the three parameters with median levels of BNP at 388 pg/mL (*N* < 100 pg/mL), median NT-pro-BNP levels at 4,328 pg/mL (*N* < 450 pg/mL), and median troponin T levels at 0.08 ng/mL (*N* < 0.1 ng/mL) (32). Similar results were found in other studies without ICU recruitment. Whittaker et al. had a mean NT-proBNP level of 788 pg/mL (174–10,548) for a norm under 100, and a mean troponin level at 45 ng/mL (8-294) for a norm under 15. Feldstein et al. reported the same trend in their 186 patients with 94/128 (73%) patients who had elevated levels of BNP and 77/153 (50%) who had elevated troponin levels (43).

## Biological Findings

### Inflammatory Markers and Hematological Abnormalities

Elevation of inflammatory markers is always present, such as erythrocyte sedimentation rate (ESR) and C-reactive protein (CRP). Especially CRP with values between 20 mg/L and up to 200 mg/dL (16, 17, 36).

Different markers of inflammation are also frequently elevated like D-dimer levels above 500 ng/mL, ferritin, but also procalcitonin levels with no bacterial infection associated, and lactate dehydrogenase (LDH). Other common findings include lymphopenia, neutrophilia, thrombocytopenia, hyponatremia, or hypoalbuminemia (13, 14, 16, 17, 19, 20, 34, 36, 45). Inflammatory cytokine levels are elevated as well. Interleukin 6 (IL-6), tumor necrosis factor alpha (TNFα), or IL10 are often elevated (47). In the study by Toubiana et al. mean IL-6 levels were 170 pg/mL (4–1,366) (45). IL6 elevation is suggested to be associated with poorer outcomes in adult patients (47).

#### SARS-CoV-2 Serology and PCR

Biological evidence SARS-CoV-2 is not required to define PIMS. Most patients have negative PCR testing for SARS-CoV-2 but most of them have positive serology. Out of 99 US patients with PIMS, Dufort and colleagues reported an RT-PCR positivity in 50/98 patients (51%) but positive serology in 76/77 patients (99%) (17). A similar result was found in the English cohort of Whittaker et al. with only 15/56 (26%) patients positive for RT-PCR but 40/46 (83%) positive for SARS-CoV-2 IgG antibody (14). In a French and Swiss study, Belhadjer et al. reported also nasopharyngeal swab PCR positive in 12 patients (34%), fecal PCR was positive in 2 patients (6%), but of 35 patients, 30 (86%) had positive antibody assays (19). Even if biological positivity for SARS-COV-2 and temporal association is not mandatory, as PIMS happens between 3 and 6 weeks after acute infection in an area where there was peak incidence, the time factor is also part of the diagnosis.

SARS-CoV-2 antibody detection can use either the spike protein (S) and/or the nucleocapsid protein (N) of the virus (48, 49). Their specificity and sensitivity can differ from one study to the other as can the type of assay used, either immunofluorescence or, more commonly, enzyme-linked immunosorbent assay (50, 51). The type of immunoglobulin studied is also of importance, as we know that IgM appear sooner than IgG and IgA, and affinity can vary (52–54). These parameters should be considered when interpreting serological results.

## Management of Patients with PIMS

PIMS is a new disease and its underlying mechanisms probably involve an excessive inflammatory response. As a new disease, its treatment sticks to clinical observation and similarities with other inflammatory syndromes.

### Supportive Care

Originally described as a life-threatening condition, most patients were treated in the pediatric intensive care unit (PICU) with a need for fluid expansion, inotropic drugs, and rarely mechanical circulatory assistance with veno-arterial extracorporeal membrane oxygenation (13, 19). With more patients being reported, the clinical spectrum appears to be wider and not always as worrisome as first thought (44).

There is no consensus on the specific and optimal treatment. Clinical studies are necessary to optimize management. Multi-organ dysfunctions are treated as they come but a specific treatment for the hyperinflammatory condition is not yet universal.

### Immunologic Treatment Approaches

#### Intravenous Immunoglobulins

Intravenous immunoglobulin (IVIG) have multiple immune effects. It confers passive immunity, anti-inflammatory effect, and immunomodulation. An IgG molecule executes its functions through humoral and cellular immunity. It can block immune complexes by binding to the Fcγ receptors, thus preventing the expansion of regulatory T lymphocytes, and also prevent cell-cell interactions by binding to cell-surface receptors (55). But IVIG shortages in supply are regularly happening and added to its cost, this might not make it available where and when it is needed (56).

As it was first recognized as a close-related syndrome to KD, PIMS was treated like KD (57). Most patients received IVIG as a first-line treatment, either 2 g/kg once or 1 g/kg twice in the first 24 h depending on fluid overload and renal function. A second dose was often given if an inflammatory state persisted, still in the hypothesis of a KD-related syndrome (13, 14, 16, 17, 21, 25, 27–29, 33, 36, 38, 45).

#### Corticosteroids

Corticosteroids are one of the most powerful anti-inflammatory drugs. They provide non-specific down-regulation of multiple pro-inflammatory cytokines like IL-1β, IL-2, IL-6, TNF-α, and IL-17. This broad ability has proved useful in autoinflammatory disorders (58). They have been used for a long time and their complex mechanisms of action are, to this day, strikingly, not fully understood. Inhibition of the vastly expressed pro-inflammatory factor nuclear factor kappa B (NF-κB) is one of the ways (59). Corticosteroids are recommended in the hyperinflammatory state related to acute respiratory distress syndrome (60) and have been used in severe COVID-19 patients with severe pneumonia (61). Corticosteroids are widely used in inflammatory conditions. As PIMS have remarkably similar features to cytokine storm syndromes, treatments used in these conditions have been proposed (13, 14, 16, 17, 21, 28, 36, 38, 45, 62).

#### Biological Treatments

On the other side of wide non-specific downregulation, a more precise approach is to target inflammatory cytokines to reduce hyper inflammation in PIMS patients. The different biological agents used were mostly anti-cytokine treatment like Anakinra, an IL1 receptor antagonist or Tocilizumab, an anti-IL6, or scarcely infliximab, an anti-TNFα (17, 32, 43).

##### Anakinra

Interleukin (IL)-1α and IL-1β bind to the IL-1 receptor type 1 and triggers an inflammatory cascade. The IL-1 receptor is present in almost every cell and is a key component of the innate immune response (63). Anakinra is an IL-1 receptor antagonist with a strong anti-inflammatory effect currently effective in several auto-inflammatory diseases and possibly in macrophage activation syndrome. Anakinra has a good safety profile even in case of acute infection (64–66). In IVIG-resistant KD, it was without major adverse events, and it showed clinical efficiency by reducing fever, as well as biological efficiency in lowering markers of inflammation, and echocardiographic features like coronary artery dilatation (67). As an extrapolation to IVIG resistant KD, anakinra has been used in PIMS (14, 16, 21, 27, 28).

##### Tocilizumab

Tocilizumab, a humanized antihuman IL-6 receptor antibody that inhibits IL-6 activity, is already an effective treatment of juvenile idiopathic arthritis and other rheumatological diseases (68–70). As IL-6 levels were elevated in many patients with PIMS, a targeted approach was conducted in several patients. However, their number is limited and there are no control groups, therefore, we cannot draw any conclusion regarding the efficacy of tocilizumab in PIMS patients (14, 16, 17, 21, 27, 29, 38, 43).

A recent study by Guaraldi et al. has shown interesting results using tocilizumab in patients with severe COVID-19 pneumonia. In a multicentre, open-label study, they included 544 of 1,351 patients admitted with severe COVID-19 pneumonia. They compared two groups, one with standard care and one with tocilizumab either intravenous (IV) or subcutaneous (SC). Their primary outcome was a composite criterion of either death or invasive mechanical ventilation. In the standard care group, 57/365 (16%) patients needed mechanical ventilation compared with 33/179 (18%) patients treated with tocilizumab (*p* = 0.41). They had 73 (20%) patients in the standard care group who died, compared with 13 (7%) (*p* < 0.0001) patients treated with tocilizumab either IV or SC. After multivariate adjustment, they showed that tocilizumab treatment was associated with a reduction in their composite outcome: mechanical ventilation or death (71). Extrapolation of these results to PIMS patients is hazardous while these patients neither had severe pulmonary involvement requiring mechanical ventilation nor commonly died.

Anti-TNFα are used widely in auto-immune diseases and have been scarcely proposed in PIMS (17).

## Pathophysiological Hypotheses

The pathogenesis of COVID-19 is not completely understood and the difference in COVID19 severity between adults and children is probably multifactorial.

### Epidemiologic Strong Points

The onset of PIMS in highly SARS-CoV2 infected regions happened between 3 and 6 weeks after COVID19 peaks, and since new COVD19 cases decreased, so have PIMS cases (15). Most patients were negative for RT-PCR testing but positive for serology testing. This time shift, coupled with the low positivity rate of direct testing, suggest a post-infectious mechanism rather than a direct viral strike. Children of African or Hispanic origin got PIMS more frequently and children of Asian origin appeared less affected. Six out of 8 patients were of African ancestry in the first report; in the Feldstein report only 19% were white non-Hispanic but 25% were black non-Hispanic and 31% were Hispanic or Latino. Dufort et al. reported 31/78 (40%) Black patients (13, 17, 43). Thus, genetic susceptibility may account for this ethnic overrepresentation in some patients.

### Heterogeneity of the Immunologic Response

#### IFN Type I Response May Have a Critical Role

Some clues suggest that PIMS is not viral-mediated but more likely to be a delayed dysregulated immune response to SARS-CoV-2. The time delay between an epidemic peak in cases and flares of PIMS cases, as well as the low number of patients positive for SARS-CoV-2 with RT-PCR, coupled with a high proportion of patients who were antibody positive, are among them. But some intrinsic, immune-related factors might have a role.

Hadjadj et al. showed that, in adult patients with severe COVID-19 disease, an impaired IFN type 1 might be responsible for the severe clinical phenotype during primary infection. They compared 50 COVID-19 patients divided into three different severity groups (compared to healthy controls) and found that severe disease progression was associated with a lower Interferon Signaling Gene (ISG) score, an impaired IFN-α production, and that it was associated with lower viral clearance (72). Trouillet-Assant and colleagues have described 26 patients with COVID-19 hospitalized in an intensive care unit (ICU) and measured their cytokine levels and IFN response. They showed that five of the patients who had no IFN-α production presented with poor evolution, as all five of them required mechanical ventilation and had a longer period in ICU (73). They did multiple time points and showed that following day 10, IL-6 stayed high whereas IFN-α went down. They concluded that this kinetics could underline that cytokine inhibitors might be used after the initial phase once IFN-α diminishes. The timing of the IFN response may be key to explaining severe COVID-19 pathogenesis.

On the other hand, outbreaks of chilblain-like lesions have been described in adolescents weeks after symptoms compatible with COVID19 (12–14). In the study by Landa et al. they described six patients with chilblain-like lesions, mostly without other coronavirus symptoms. Only very few of them had a cough, fever, or nasal congestion 3–4 weeks before presentation. Two had a positive PCR test weeks before. Mazzotta et al. reported a case of a 13 years old boy 6 weeks after the disease peak in Italy that presented with chilblain. Another Italian study reported 63 patients with chilblain-like lesions (74). Chilblain is also found in vasculitis and diseases presenting a pathological excess of interferon type 1 signaling like type 1 interferonopathies or systemic lupus (75). It could be that both phenotypes (severe pneumonia on one side, chilblain, and PIMS on the other side) are two ends of the spectrum with a deregulated response resulting in a severe onset in some individuals and a post-infectious inflammatory syndrome in others. Laboratory findings and more studies are needed to explore this hypothesis.

A delay in IFN response with a low viral clearance and a persistent and inadequate IFN response has already been hypothesized (76, 77). In this perspective, the study by Bronte et al. showing the use of baricitinib, a JAK1-2 inhibitor, blocking the JAK/STAT pathway is relevant (78). The STAT3 pathway is also relevant for the production of some cytokines like IL-6 and IL-10.

#### Other Hypotheses

In their study, Dioro et al. imply that PIMS and severe COVID-19 are two different entities, both caused by SARS-CoV-2. They analyzed 20 patients divided into three groups: 9 considered as “severe COVID-19” hospitalized in the pediatric ICU, 5 patients with “minimal COVID-19” with no respiratory support needed, among different criteria, and 6 patients with PIMS. They measured different cytokines levels to identify which ones could discriminate between the three conditions. They used a 10 cytokine panel (IFN-γ, IL-1β, IL-2, IL-4, IL-6, IL-8, IL-10, IL-12p70, IL-13, and TNF-α) but excluded five that remained low in the three different groups (IL-1β, IL-2, IL-4, IL-12p70, and IL-13). They found that the sum of TNF-α and IL10 combined could distinguish patients with PIMS apart from the patients with severe COVID-19, as they had higher levels than severe COVID-19 patients (79).

Interferon gamma-induced protein 10 (IP-10) also has been suggested as part of the distinction for patients at risk. Also known as chemokine (C-X-C motif) ligand 10 (CXCL10), IP-10 secretion is mainly regulated by IFN-γ (80). In their study, Laing et al. showed that patients with elevated levels of IP-10, IL6, and IL10 on their first bleed were more at risk of a worsened condition in the 1st week than patients with normal or not so high elevation. IP-10 producing cells were depleted in these patients, and even though IP-10 levels had some correlation with IFN-γ and IFN-α levels, they suggested that IP-10 elevation might be caused by other virus-associated pathways (47). In a preprint, Yang et al. discussed the elevations of IP-10 in severe patients as well (81). The impact of IL10 and IP10 on PIMS patients requires further investigation.

A recent study reported a patient with haploinsufficiency in SOCS1 and autoimmune thrombocytopenia, who displayed a severe inflammatory response to SARS-CoV2. SOCS1 is a key regulatory molecule inhibiting cytokine signaling, especially type I/II interferons (82). Even though the phenotype was not completely reminiscent of PIMS, as it developed much faster than other patients, cytokine hyperresponsiveness may explain part of the PIMS pathogenesis.

### Virus-Related Factors

#### Variability of ACE2 Expression

SARS-CoV-2 is a part of the β coronavirus genus along with SARS-CoV and Middle East Respiratory Syndrome. Its transmission between humans is mainly through dispersed droplets during close contact or through a contaminated surface (83). Virus entrance in the cell consists of biding to the angiotensin-converting enzyme 2 (ACE2), which expression is higher in lung cells, cardiomyocytes, vascular endothelium, alveolar cells, and some immune cells (84, 85). ACE2 expression variability between individuals might explain the different courses of the disease, from asymptomatic to severe pneumonia. ACE2 expression is at its peak in children and then decreases over the years. Some conditions like type 2 diabetes or hypertension lower its expression even more. These patients have been described to be more susceptible to severe COVID-19 pneumonia, thus, lower ACE2 expression might be associated with a higher risk of severe disease (86). ACE2 is part of the ACE2—angiotensin-(1–7)—Mas system, which balances the inflammatory effects of the ACE—angiotensin-2 axis (87). ACE2 processes angiotensin-2 into angiotensin1-7, which in turn, is responsible for downregulating pro-inflammatory events. A higher number of ACE2 expression when the infection arises might be beneficial. Indeed, a higher density of ACE2 could prove beneficial by keeping angiotensin1-7 levels relatively high compared to angiotensin2 and its pro-inflammatory features. This could be one of the reasons children can have COVID-19 but in less severe forms than the adults, and also why they are not spreading the virus as much as we initially thought.

#### Exposure to Common Coronaviruses

As we just discussed one hypothesis that could confer a more balanced immune response in children, another one is that recent infections to common coronaviruses in children allow them to have some immunity to SARS-CoV-2 (88). Most children have exposition to common coronaviruses before the age of 5 and have effective seroconversion, then, antibody levels diminish over the years (89). Cross-reactivity between common coronaviruses and SARS-COV-2 exists. It was observed that anti-seasonal common coronaviruses titers are increased in the serum of SARS-CoV-2 patients (90). This could be part of the explanation of why children are less infected but some present delayed hyperinflammatory response as cross-reactivity might also increase inflammation response.

#### Similarities With Toxic Shock Syndrome and the Super Antigen Theory

Toxic shock syndrome (TSS) is a syndrome characterized by shock, cytokine storm, and multi-organ involvement possibly leading to multiple organ failures and death. It is secondary to an infection by *Staphylococcus aureus* or *Streptococcus pyogenes* toxin-producing specific strains. Toxins have a superantigen activity and are responsible for an excessive activation of T cells (91). Superantigen activity differs from classical antigen activity and leads to an activation of a larger number of T cells, as they do not bind to the same peptide-binding area, thus generating a larger cytokine storm (91).

PIMS shares some clinical and biological similarities with TSS such as high fever, multiorgan involvement, and elevated inflammatory markers, leading to hypotension and shock (92, 93). In a retrospective study including 58 patients with TSS, vomiting was present in 44 (75.9%) patients and diarrhea in 23 (39.7%) patients. All had hypotension, multiorgan involvement, and ICU treatment (94). Another retrospective study in Australia in 62 cases found that 46 (74%) of cases were admitted to an intensive care unit and 44 (71%) required inotropic support (91).

In a preprint study, Cheng and colleagues found, using *in silico* modeling and analysis, that SARS-CoV-2 has a superantigen-like motif near its S1/S2 cleavage site. This motif has similarities to the staphylococcal enterotoxins B (SEB) superantigen (SAg) one that is responsible for TSS. SEB causes a massive inflammatory cascade which results in high levels of proinflammatory cytokines like IFN-γ, IL-2, IL-1, and TNFα (95). Because this cytokine storm is quite similar to the one we see in PIMS patients, as we discussed with high levels of TNFα, the authors suggested that PIMS is a consequence of a superantigen-like activity of the SARS-CoV-2 S protein (96). In a second part of the article, they wrote that a rare mutation, D839Y/E, found in a SARS-CoV-2 strain from Europe, could contribute to increasing the interaction between the virus and TCR, thus enhancing the pro-inflammatory effect and could also explain the differences between European and North American reports of PIMS compared to Asian reports.

## SARS-COV-2 Infection in Patients With Rheumatic Diseases

Children with rheumatological and musculoskeletal diseases (RMDs) are historically more susceptible to infections either because of their disease or their treatment (97, 98). Disease-modifying anti-rheumatic drugs like methotrexate have immunomodulatory effects that affect a host response to an infection (99). Viral infections, and especially respiratory infections, are the ones for which there is a higher risk (100).

In this regard, specific attention was put on adults and children with RMDs.

### Adult Patients With Rheumatic Diseases

Data on COVID-19 severity and adult patients with RMDs are conflicting.

In a cross-sectional study in Tuscany, 458 patients had a phone interview to obtain information on either presence of symptoms compatible with COVID-19 or confirmed infection. Seventy-four percent were female and the median age was 56 years. Among the 458 patients, 13 declared symptoms compatible with COVID-19 but only one was positive out of seven who underwent nasopharyngeal swab. She developed severe complications and had to be admitted to ICU. Only 30 patients (6.6%) had active disease at the time of the study. The predominant disease was systemic lupus erythematosus with 117 (25.6%) patients. Among the different on-going treatments, 56% were treated with corticosteroids in the whole population compared to 69.2% (9/13) in the symptoms group. Among all patients, 44% were receiving DMARDs, 8/13 in the symptoms group, with 3 of them having hydroxychloroquine. Forty-one percent were on biologic treatment, 7/13 in the symptoms groups, with 4 receiving anti-TNFα treatment and 3 receiving belimumab, an anti-BAFF treatment. The authors concluded that the prevalence of SARS-CoV-2 infection in their cohort was similar to that observed in Tuscany at the time of the study with 0.22% compared to 0.20% (101). Another study done in Italy found similar results with 1,525 patients with RMDs. Of those 1,525 patients, 117 (8%) presented with symptoms that were compatible with COVID-19 (102). On the other hand, in the same journal issue, a retrospective study from China looked at the susceptibility to have COVID-19 in a cohort of 6,228 patients with RMDs. They wanted to evaluate the risk for a patient with RMDs to have COVID-19 when exposed and used family members without RMDs as a comparative group. Among their cohort of RMDs patients, they searched for patients with a confirmed COVID-19 exposure in their families and found 42 families. Among those families, they compared the number of COVID-19 patients with RMDs to other COVID-19 family members without RMD. They found that within these 42 families, COVID-19 was diagnosed in 27 (63%) of 43 patients with an RMD and 28 (34%) of 83 family members without RMD. In other words, patients with RMDs had an odds ratio (OR) of 2.68 (*p* = 0.023) of developing COVID-19 compared to their family members without RMD. The authors concluded that people with RMDs might be at greater risk of infection than the general population However, only 27 out of their 6,228 patients in their cohort had a diagnosis of COVID-19.

Finally, an international registry for patients with rheumatic disease reported 600 cases from 40 countries. Forty-six percent (277/600 cases) were hospitalized and 55 (9%) died. They looked at different parameters to highlight risk factors associated with hospitalization or death. They found with multivariable adjustment models that a corticoid treatment with a prednisone equivalent dose superior to 10 mg/day was associated with a higher risk of hospitalization (odds ratio (OR) 2.05). On the other hand, anti-TNFα treatment was protective of hospitalization with an OR of 0.40. No other treatment was associated with a modified OR for hospitalization, especially hydroxychloroquine or DMARDs (11).

### Children With Rheumatic Diseases

There are little data on children with rheumatological diseases and COVID-19. A web-based survey study of patients under 23 years old with auto-inflammatory diseases in Turkey found that among 404 patients (217 female) that answered the survey, only 24 (5.9%) had been admitted to the hospital for COVID-19 suspicion. Seven of them had a positive rhino-pharyngeal swab and all of them recovered completely. The different auto-inflammatory diseases were Familial Mediterranean Fever (FMF) in 364 (90%) of the patients, the next most frequent was periodic fever aphthous stomatitis pharyngitis and adenitis (PFAPA) in 14 (3.5%) patients. The overwhelming majority of patients with FMF is part of the reason why colchicine treatment was taken by 375 (93%) of the patients. Other treatments included biological molecules for 48 (11.8%) patients. Biological drugs were canakinumab for 35 patients, anakinra for 5 patients, etanercept, adalimumab, and tocilizumab, each for one patient. Among patients receiving biologic treatment, none of them had a positive PCR test or any symptoms (103).

As an autoinflammatory condition does not seem to be of higher risk, we looked at the other end of the immune spectrum with immune deficiency. A recent study included 585 cases of SARS-CoV-2 infection in children from 21 European countries, between the 1st and 24th of April. Gotzinger et al. (104) reported that among those, only 29 (5%) individuals were receiving immunosuppressive treatment at the time of infection. Among those 29, only three needed admissions to ICU (10%) which is similar to the percentage of patients admitted in the ICU in the entire study with 48/582 (9%). We had no information on immune treatment before infection. They reported three (1%) patients with a previously diagnosed immune deficiency (common variable immunodeficiency, congenital neutropenia, and Schimke syndrome) and none of them was admitted to the ICU. Twenty-five (4%) were receiving chemotherapy at the time of their diagnosis or had received chemotherapy in the preceding 6 months, only 2/25 (8%) went to the ICU. Among the patients who received immunomodulatory treatment before the infection or had a pre-existing medical condition with immune deficiency, this study suggests there is not a major higher risk of severe COVID-19.

These different studies, as well as different unpublished experiences and discussions among physicians attending patients with RMDs, suggest that adult patients and, to an extent, pediatric patients with rheumatic diseases do not seem to be at a higher risk of severe COVID-19 and PIMS.

## Conclusion

Pediatric Inflammatory Multisystem Syndrome (PIMS) is related to SARS-CoV-2 infection. Clinical symptoms are wide and non-specific but always feature high fever, multiorgan involvement, and strong biological inflammation. Pathophysiology of PIMS, which is above all an excess of an inflammatory response, justifies the use of immunomodulatory treatments. Patients with severe complications require symptomatic treatment among which the treatment of cardiac failure with inotropes has a special place and allows rapid recovery. Children receiving immunomodulatory treatment for chronic rheumatic and inflammatory diseases do not seem at a greater risk of PIMS but evidence and data are scarce.

## Author Contributions

AS wrote the first draft upon AB and IK-P proposition. The minireview was edited and adapted by AB and IK-P. All authors agreed with the final document.

## Conflict of Interest

The authors declare that the research was conducted in the absence of any commercial or financial relationships that could be construed as a potential conflict of interest.

## Abbreviations

BNP: B-type natriuretic peptide
COVID-19: Coronavirus disease 19
CRP: C-reactive protein
ECG: electrocardiogram
ESR: erythrocyte sedimentation rate
ICU: intensive care unit
IFN: interferon
IL: interleukin
IP-10: Interferon gamma-induced protein 10
ISG: Interferon Signaling Gene
IVIG: Intravenous immunoglobulin
KD: Kawasaki Disease
MIS-C: Multi Inflammatory Syndrome in Children
PIMS: Pediatric Inflammatory Multisystem Syndrome
proBNP: pro-B-type natriuretic peptide
RMDs: rheumatological and musculoskeletal diseases
SARS-CoV-2: Severe Acute Respiratory Syndrome Coronavirus type 2
TNF: Tumor Necrosis Factor
TSS: Toxic Shock Syndrome.

## References

[1] COVID-19 Map Johns Hopkins Coronavirus Resource Center. Available online at: https://coronavirus.jhu.edu/map.html (accessed September 12, 2020).

[2] Cai J Xu J Lin D Yang Z Xu L Qu Z. A case series of children with 2019 novel coronavirus infection: clinical and epidemiological features. Clin Infect Dis. (2020) 71:1547–51. 10.1093/cid/ciaa198 32112072 PMC7108143

[3] Xia W Shao J Guo Y Peng X Li Z Hu D. Clinical and CT features in pediatric patients with COVID-19 infection: different points from adults. Pediatr Pulmonol. (2020) 55:1169–74. 10.1002/ppul.24718 32134205 PMC7168071

[4] Wei M Yuan J Liu Y Fu T Yu X Zhang Z-J. Novel coronavirus infection in hospitalized infants under 1 year of age in China. JAMA. (2020) 323:1313–4. 10.1001/jama.2020.2131 32058570 PMC7042807

[5] Lu X Zhang L Du H Zhang J Li YY Qu J SARS-CoV-2 infection in children. N Engl J Med. (2020) 382:1663–5. 10.1056/NEJMc2005073 32187458 PMC7121177

[6] Wu Z McGoogan JM. Characteristics of and important lessons from the coronavirus disease 2019 (COVID-19) outbreak in china: summary of a report of 72, 314 cases from the chinese center for disease control and prevention. JAMA. (2020). 323:1239–42. 10.1001/jama.2020.2648 32091533

[7] COVID-19 Available online at: https://qap.ecdc.europa.eu/public/extensions/COVID-19/COVID-19.html (accessed August 21, 2020).

[8] Canada PHA of Epidemiological summary of COVID-19 cases in Canada (2020). Available online at: https://health-infobase.canada.ca/covid-19/epidemiological-summary-covid-19-cases.html#a5 (accessed August 21, 2020).

[9] Epidemiology Working Group for NCIP Epidemic Response, Chinese Center for Disease Control and Prevention. [The epidemiological characteristics of an outbreak of 2019 novel coronavirus diseases (COVID-19) in China]. Zhonghua Liu Xing Bing Xue Za Zhi. (2020) 41:145–51. 10.3760/cma.j.issn.0254-6450.2020.02.003 32064853

[10] Gianfrancesco M Yazdany J Robinson PC. Epidemiology and outcomes of novel coronavirus 2019 in patients with immune-mediated inflammatory diseases. Curr Opin Rheumatol. (2020) 32:434–40. 10.1097/BOR.0000000000000725 32675715

[11] Gianfrancesco M Hyrich KL Al-Adely S Carmona L Danila MI Gossec L. Characteristics associated with hospitalisation for COVID-19 in people with rheumatic disease: data from the COVID-19 global rheumatology alliance physician-reported registry. Ann Rheum Dis. (2020) 79:859–66. 10.1136/annrheumdis-2020-217871 32471903 PMC7299648

[12] Kilian A Chock YP Huang IJ Graef ER Upton LA Khilnani A. Acute respiratory viral adverse events during use of antirheumatic disease therapies: a scoping review. Semin Arthritis Rheum. (2020) 50:1191–201. 10.1016/j.semarthrit.2020.07.007 32931985 PMC7832282

[13] Riphagen S Gomez X Gonzalez-Martinez C Wilkinson N Theocharis P. Hyperinflammatory shock in children during COVID-19 pandemic. Lancet. (2020) 395:1607–8. 10.1016/S0140-6736(20)31094-1 32386565 PMC7204765

[14] Whittaker E Bamford A Kenny J Kaforou M Jones CE Shah P. Clinical characteristics of 58 children with a pediatric inflammatory multisystem syndrome temporally associated with SARS-CoV-2. JAMA. (2020) 324:259–69. 10.1001/jama.2020.10369 32511692 PMC7281356

[15] Belot A Antona D Renolleau S Javouhey E Hentgen V Angoulvant F. SARS-CoV-2-related paediatric inflammatory multisystem syndrome, an epidemiological study, France, 1 March to 17 May 2020. Euro Surveill Bull Eur Sur Mal Transm Eur Commun Dis Bull. (2020) 25:2001010. 10.2807/1560-7917.ES.2020.25.22.2001010 32524957 PMC7336112

[16] Pouletty M Borocco C Ouldali N Caseris M Basmaci R Lachaume N. Paediatric multisystem inflammatory syndrome temporally associated with SARS-CoV-2 mimicking Kawasaki disease (Kawa-COVID-19): a multicentre cohort. Ann Rheum Dis. (2020) 79:999–1006. 10.1136/annrheumdis-2020-217960 32527868 PMC7299653

[17] Dufort EM Koumans EH Chow EJ Rosenthal EM Muse A Rowlands J. Multisystem inflammatory syndrome in children in New York State. N Engl J Med. (2020) 383:347–58. 10.1056/NEJMoa2021756 32598830 PMC7346766

[18] Perez-Toledo M Faustini SE Jossi SE Shields AM Kanthimathinathan HK Allen JD. Serology confirms SARS-CoV-2 infection in PCR-negative children presenting with paediatric inflammatory multi-system syndrome. MedRxiv [Preprint]. (2020). 10.1101/2020.06.05.20123117 32577677 PMC7302282

[19] Belhadjer Z Méot M Bajolle F Khraiche D Legendre A Abakka S. Acute heart failure in multisystem inflammatory syndrome in children (MIS-C) in the context of global SARS-CoV-2 pandemic. Circulation. (2020) 142:429–36. 10.1161/CIRCULATIONAHA.120.048360 32418446

[20] Verdoni L Mazza A Gervasoni A Martelli L Ruggeri M Ciuffreda M. An outbreak of severe Kawasaki-like disease at the Italian epicentre of the SARS-CoV-2 epidemic: an observational cohort study. Lancet. (2020) 395:1771–8. 10.1016/S0140-6736(20)31103-X 32410760 PMC7220177

[21] Grimaud M Starck J Levy M Marais C Chareyre J Khraiche D. Acute myocarditis and multisystem inflammatory emerging disease following SARS-CoV-2 infection in critically ill children. Ann Intensive Care. (2020) 10:69. 10.1186/s13613-020-00690-8 32488505 PMC7266128

[22] Ng KF Kothari T Bandi S Bird PW Goyal K Zoha M. COVID-19 multisystem inflammatory syndrome in three teenagers with confirmed SARS-CoV-2 infection. J Med Virol. (2020). 10.1002/jmv.26206 32568434 PMC7362099

[23] Blondiaux E Parisot P Redheuil A Tzaroukian L Levy Y Sileo C Cardiac MRI of children with Multisystem Inflammatory Syndrome (MIS-C) associated with COVID-19: case series. Radiology. (2020) 297:E283–8. 10.1148/radiol.2020202288 32515676 PMC7294821

[24] Capone CA Subramony A Sweberg T Schneider J Shah S Rubin L. Characteristics, cardiac involvement, and outcomes of multisystem inflammatory syndrome of childhood associated with severe acute respiratory syndrome coronavirus 2 infection. J Pediatr. (2020) 224:141–5. 10.1016/j.jpeds.2020.06.044 32553873 PMC7293762

[25] Jones VG Mills M Suarez D Hogan CA Yeh D Segal JB. COVID-19 and Kawasaki disease: novel virus and novel case. Hosp Pediatr. (2020) 10:537–40. 10.1542/hpeds.2020-0123 32265235

[26] Ouldali N Pouletty M Mariani P Beyler C Blachier A Bonacorsi S. Emergence of Kawasaki disease related to SARS-CoV-2 infection in an epicentre of the French COVID-19 epidemic: a time-series analysis. Lancet Child Adolesc Health. (2020) 4:662–8. 10.1016/S2352-4642(20)30175-9 32622376 PMC7332278

[27] Waltuch T Gill P Zinns LE Whitney R Tokarski J Tsung JW. Features of COVID-19 post-infectious cytokine release syndrome in children presenting to the emergency department. Am J Emerg Med. (2020). 10.1016/j.ajem.2020.05.058. [Epub ahead of print]. 32471782 PMC7255141

[28] Miller J Cantor A Zachariah P Ahn D Martinez M Margolis K. Gastrointestinal symptoms as a major presentation component of a novel multisystem inflammatory syndrome in children (MIS-C) that is related to COVID-19: a single center experience of 44 cases. Gastroenterology. (2020) 159:1571–4.e2. 10.1053/j.gastro.2020.05.079 32505742 PMC7270806

[29] Balasubramanian S Nagendran TM Ramachandran B Ramanan AV. Hyper-inflammatory syndrome in a child with COVID-19 treated successfully with intravenous immunoglobulin and tocilizumab. Indian Pediatr. (2020) 57:681–3. 10.1007/s13312-020-1901-z 32393681 PMC7387261

[30] Rivera-Figueroa EI Santos R Simpson S Garg P. Incomplete kawasaki disease in a child with Covid-19. Indian Pediatr. (2020) 57:680–1. 10.1007/s13312-020-1900-0 32393680 PMC7387257

[31] Riollano-Cruz M Akkoyun E Briceno-Brito E Kowalsky S Reed J Posada R. Multisystem inflammatory syndrome in children related to COVID-19: a New York City experience. J Med Virol. (2020). 10.1002/jmv.26224. [Epub ahead of print]. 32584487 PMC7361761

[32] Kaushik S Aydin SI Derespina KR Bansal PB Kowalsky S Trachtman R. Multisystem inflammatory syndrome in children associated with severe acute respiratory syndrome coronavirus 2 infection (MIS-C): a multi-institutional study from New York City. J Pediatr. (2020) 224:24–9. 10.1016/j.jpeds.2020.06.045 32553861 PMC7293760

[33] Chiotos K Bassiri H Behrens EM Blatz AM Chang J Diorio C. Multisystem inflammatory syndrome in children during the coronavirus 2019 Pandemic: a case series. J Pediatr Infect Dis Soc. (2020) 9:393–8. 10.1093/jpids/piaa069 32463092 PMC7313950

[34] Cheung EW Zachariah P Gorelik M Boneparth A Kernie SG Orange JS. Multisystem inflammatory syndrome related to COVID-19 in previously healthy children and adolescents in New York City. JAMA. (2020) 324:294–6. 10.1001/jama.2020.10374 32511676 PMC7281352

[35] Schupper AJ Yaeger KA Morgenstern PF. Neurological manifestations of pediatric multi-system inflammatory syndrome potentially associated with COVID-19. Childs Nerv Syst. (2020) 36:1579–80. 10.1007/s00381-020-04755-8 32583150 PMC7314616

[36] Licciardi F Pruccoli G Denina M Parodi E Taglietto M Rosati S. SARS-CoV-2-Induced Kawasaki-like hyperinflammatory syndrome: a novel COVID phenotype in children. Pediatrics. (2020) 146:e20201711. 10.1542/peds.2020-1711 32439816

[37] Hameed S Elbaaly H Reid CEL Santos RMF Shivamurthy V Wong J. Spectrum of imaging findings on chest radiographs, US, CT, and MRI images in Multisystem Inflammatory Syndrome in Children (MIS-C) associated with COVID-19. Radiology. (2020). 10.1148/radiol.2020202543. [Epub ahead of print]. 32584166 PMC7769068

[38] Greene AG Saleh M Roseman E Sinert R. Toxic shock-like syndrome and COVID-19: a case report of multisystem inflammatory syndrome in children (MIS-C). Am J Emerg Med. (2020). 10.1016/j.ajem.2020.05.117. [Epub ahead of print]. 32532619 PMC7274960

[39] Sadiq M Aziz OA Kazmi U Hyder N Sarwar M Sultana N. Multisystem inflammatory syndrome associated with COVID-19 in children in Pakistan. Lancet Child Adolesc Health. (2020) 4:e36–7. 10.1016/S2352-4642(20)30256-X 32791052 PMC7417160

[40] Multisystem inflammatory syndrome in children and adolescents with COVID-19 Available online at: https://www.who.int/publications-detail-redirect/multisystem-inflammatory-syndrome-in-children-and-adolescents-with-covid-19 (accessed August 21, 2020).

[41] Guidance- Paediatric multisystem inflammatory syndrome temporally associated with COVID-19 (PIMS) RCPCH. Available online at: https://www.rcpch.ac.uk/resources/guidance-paediatric-multisystem-inflammatory-syndrome-temporally-associated-covid-19-pims (accessed August 21, 2020).

[42] HAN Archive - 00432 | Health Alert Network (HAN). (2020). Available online at: https://emergency.cdc.gov/han/2020/han00432.asp (accessed August 21, 2020).

[43] Feldstein LR Rose EB Horwitz SM Collins JP Newhams MM Son MBF. Multisystem inflammatory syndrome in U.S. children and adolescents. N Engl J Med. (2020) 383:334–46. 10.1056/NEJMoa2021680 32598831 PMC7346765

[44] Godfred-Cato S Bryant B Leung J Oster ME Conklin L Abrams J. COVID-19-associated multisystem inflammatory syndrome in children - United States, March-July 2020. MMWR Morb Mortal Wkly Rep. (2020) 69:1074–80. 10.15585/mmwr.mm6932e2 32790663 PMC7440126

[45] Toubiana J Poirault C Corsia A Bajolle F Fourgeaud J Angoulvant F. Kawasaki-like multisystem inflammatory syndrome in children during the covid-19 pandemic in Paris, France: prospective observational study. BMJ. (2020) 369:m2094. 10.1136/bmj.m2094 32493739 PMC7500538

[46] Webb K Abraham DR Faleye A McCulloch M Rabie H Scott C. Multisystem inflammatory syndrome in children in South Africa. Lancet Child Adolesc Health. (2020) 4:e38. 10.1016/S2352-4642(20)30272-8 32835654 PMC7442431

[47] Laing AG Lorenc A del Molino del Barrio I Das A Fish M Monin L A dynamic COVID-19 immune signature includes associations with poor prognosis. Nat Med. (2020) 26:1623–35. 10.1038/s41591-020-1038-6 32807934

[48] Zhao J Yuan Q Wang H Liu W Liao X Su Y. Antibody responses to SARS-CoV-2 in patients of novel coronavirus disease 2019. Clin Infect Dis. (2020) 71:2027–34. 10.1093/cid/ciaa344 32221519 PMC7184337

[49] Guo L Ren L Yang S Xiao M Chang D Yang F. Profiling early humoral response to diagnose novel coronavirus disease (COVID-19). Clin Infect Dis. (2020) 71:778–85. 10.1093/cid/ciaa310 32198501 PMC7184472

[50] Grzelak L Temmam S Planchais C Demeret C Tondeur L Huon C. A comparison of four serological assays for detecting anti-SARS-CoV-2 antibodies in human serum samples from different populations. Sci Transl Med. (2020) 12:eabc3101. 10.1126/scitranslmed.abc3103 32817357 PMC7665313

[51] Tuaillon E Bolloré K Pisoni A Debiesse S Renault C Marie S. Detection of SARS-CoV-2 antibodies using commercial assays and seroconversion patterns in hospitalized patients. J Infect. (2020) 81:e39–45. 10.1016/j.jinf.2020.05.077 32504735 PMC7834649

[52] Wölfel R Corman VM Guggemos W Seilmaier M Zange S Müller MA. Virological assessment of hospitalized patients with COVID-2019. Nature. (2020) 581:465–9. 10.1038/s41586-020-2196-x 32235945

[53] To KK-W Tsang OT-Y Leung W-S Tam AR Wu T-C Lung DC. Temporal profiles of viral load in posterior oropharyngeal saliva samples and serum antibody responses during infection by SARS-CoV-2: an observational cohort study. Lancet Infect Dis. (2020) 20:565–74. 10.1016/S1473-3099(20)30196-1 32213337 PMC7158907

[54] Amanat F Stadlbauer D Strohmeier S Nguyen THO Chromikova V McMahon M. A serological assay to detect SARS-CoV-2 seroconversion in humans. Nat Med. (2020) 26:1033–6. 10.1038/s41591-020-0913-5 32398876 PMC8183627

[55] Schwab I Nimmerjahn F Intravenous immunoglobulin therapy: how does IgG modulate the immune system? Nat Rev Immunol. (2013) 13:176–89. 10.1038/nri3401 23411799

[56] Research C for BE and Information About Immune Globulin (Human) Product Shortage FDA. (2019). Available online at: https://www.fda.gov/vaccines-blood-biologics/safety-availability-biologics/information-about-immune-globulin-human-product-shortage (accessed November 1, 2020).

[57] de Graeff N Groot N Ozen S Eleftheriou D Avcin T Bader-Meunier B. European consensus-based recommendations for the diagnosis and treatment of Kawasaki disease - the SHARE initiative. Rheumatol Oxf Engl. (2019) 58:672–82. 10.1093/rheumatology/key344 30535127

[58] Hashkes PJ Laxer RM Simon A Textbook of Autoinflammation. Springer International Publishing (2019). 10.1007/978-3-319-98605-0

[59] Sedwick C Wanted: a new model for glucocorticoid receptor transactivation and transrepression. PLoS Biol. (2014) 12:e1001814 10.1371/journal.pbio.1001814 24642535 PMC3958365

[60] Annane D Pastores SM Rochwerg B Arlt W Balk RA Beishuizen A. Guidelines for the diagnosis and management of critical illness-related corticosteroid insufficiency (CIRCI) in critically ill patients (Part I): Society of Critical Care Medicine (SCCM) and European Society of Intensive Care Medicine (ESICM) 2017. Intensive Care Med. (2017) 43:1751–63. 10.1007/s00134-017-4919-5 28940011

[61] Crosby JC Heimann MA Burleson SL Anzalone BC Swanson JF Wallace DW. COVID-19: a review of therapeutics under investigation. J Am Coll Emerg Physicians Open. (2020) 1:231–7. 10.1002/emp2.12081 32838367 PMC7262361

[62] Henderson LA Canna SW Schulert GS Volpi S Lee PY Kernan KF. On the alert for cytokine storm: immunopathology in COVID-19. Arthritis Rheumatol. (2020) 72:1059–63. 10.1002/art.41285 32293098 PMC7262347

[63] Dinarello CA. Overview of the IL-1 family in innate inflammation and acquired immunity. Immunol Rev. (2018) 281:8–27. 10.1111/imr.12621 29247995 PMC5756628

[64] Eloseily EM Weiser P Crayne CB Haines H Mannion ML Stoll ML. Benefit of anakinra in treating pediatric secondary hemophagocytic lymphohistiocytosis. Arthritis Rheumatol Hoboken NJ. (2020) 72:326–34. 10.1002/art.41103 31513353

[65] Shakoory B Carcillo JA Chatham WW Amdur RL Zhao H Dinarello CA. Interleukin-1 receptor blockade is associated with reduced mortality in sepsis patients with features of macrophage activation syndrome: reanalysis of a prior phase III trial. Crit Care Med. (2016) 44:275–81. 10.1097/CCM.0000000000001402 26584195 PMC5378312

[66] Kuemmerle-Deschner JB Gautam R George AT Raza S Lomax KG Hur P. Systematic literature review of efficacy/effectiveness and safety of current therapies for the treatment of cryopyrin-associated periodic syndrome, hyperimmunoglobulin D syndrome and tumour necrosis factor receptor-associated periodic syndrome. RMD Open. (2020) 6:e001227. 10.1136/rmdopen-2020-001227 32723831 PMC7722275

[67] Kone-Paut I Tellier S Belot A Brochard K Guitton C Marie I. Open label, phase II study with anakinra in intravenous immunoglobulin-resistant kawasaki disease. Arthritis Rheumatol. (2020). 10.1002/art.41481. [Epub ahead of print]. 32779863

[68] Barone P Pignataro R Garozzo MT Leonardi S. IL-6 blockers in systemic onset juvenile idiopathic arthritis. Immunotherapy. (2016) 8:79–87. 10.2217/imt.15.104 26642378

[69] Machado SH Xavier RM. Safety of tocilizumab in the treatment of juvenile idiopathic arthritis. Expert Opin Drug Saf. (2017) 16:493–500. 10.1080/14740338.2017.1303479 28277841

[70] Katsicas MM Russo R. Biologic agents in juvenile spondyloarthropathies. Pediatr Rheumatol Online J. (2016) 14:17. 10.1186/s12969-016-0076-6 26968522 PMC4788890

[71] Guaraldi G Meschiari M Cozzi-Lepri A Milic J Tonelli R Menozzi M. Tocilizumab in patients with severe COVID-19: a retrospective cohort study. Lancet Rheumatol. (2020) 2:e474–84. 10.1016/S2665-9913(20)30285-X 32835257 PMC7314456

[72] Hadjadj J Yatim N Barnabei L Corneau A Boussier J Smith N. Impaired type I interferon activity and inflammatory responses in severe COVID-19 patients. Science. (2020) 369:718–24. 10.1126/science.abc6027 32661059 PMC7402632

[73] Trouillet-Assant S Viel S Gaymard A Pons S Richard J-C Perret M. Type I IFN immunoprofiling in COVID-19 patients. J Allergy Clin Immunol. (2020) 146:206–8.e2. 10.1016/j.jaci.2020.04.029 32360285 PMC7189845

[74] Piccolo V Neri I Filippeschi C Oranges T Argenziano G Battarra VC. Chilblain-like lesions during COVID-19 epidemic: a preliminary study on 63 patients. J Eur Acad Dermatol Venereol. (2020) 34:e291–3. 10.1111/jdv.16526 32330334 PMC7267498

[75] Papa R Volpi S Gattorno M. Type I interferonopathies: cutaneous vasculopathy, chilblains, panniculitis-induced lipodystrophyand others skin manifestations. G Ital Dermatol E Venereol. (2020). 10.23736/S0392-0488.20.06709-7. [Epub ahead of print]. 32618445

[76] Park A Iwasaki A. Type I and type III interferons - induction, signaling, evasion, and application to combat COVID-19. Cell Host Microbe. (2020) 27:870–8. 10.1016/j.chom.2020.05.008 32464097 PMC7255347

[77] Rowley AH. Understanding SARS-CoV-2-related multisystem inflammatory syndrome in children. Nat Rev Immunol. (2020) 20:453–4. 10.1038/s41577-020-0367-5 32546853 PMC7296515

[78] Bronte V Ugel S Tinazzi E Vella A De Sanctis F Canè S. Baricitinib restrains the immune dysregulation in patients with severe COVID-19. J Clin Invest. (2020) 141772. 10.1101/2020.06.26.20135319. [Epub ahead of print]. 32809969 PMC8016181

[79] Diorio C Henrickson SE Vella LA McNerney KO Chase JM Burudpakdee C. Multisystem inflammatory syndrome in children and COVID-19 are distinct presentations of SARS-CoV-2. J Clin Invest. (2020) 130:5967–75. 10.1172/JCI140970 32730233 PMC7598044

[80] Antonelli A Ferrari SM Giuggioli D Ferrannini E Ferri C Fallahi P Chemokine (C-X-C motif) ligand (CXCL)10 in autoimmune diseases. Autoimmun Rev. (2014) 13:272–80. 10.1016/j.autrev.2013.10.010 24189283

[81] Yang Y Shen C Li J Yuan J Yang M Wang F Exuberant elevation of IP-10, MCP-3 and IL-1ra during SARS-CoV-2 infection is associated with disease severity and fatal outcome. medRxiv [Preprint]. (2020). 10.1101/2020.03.02.20029975

[82] Lee PY Platt CD Weeks S Grace RF Maher G Gauthier K. Immune dysregulation and Multisystem Inflammatory Syndrome in Children (MIS-C) in individuals with haploinsufficiency of SOCS1. J Allergy Clin Immunol. (2020) 146:1194–200.e1. 10.1016/j.jaci.2020.07.033 32853638 PMC7445138

[83] Woo PCY Huang Y Lau SKP Yuen K-Y. Coronavirus genomics and bioinformatics analysis. Viruses. (2010) 2:1804–20. 10.3390/v2081803 21994708 PMC3185738

[84] Hamming I Timens W Bulthuis MLC Lely AT Navis GJ van Goor H. Tissue distribution of ACE2 protein, the functional receptor for SARS coronavirus. A first step in understanding SARS pathogenesis. J Pathol. (2004) 203:631–7. 10.1002/path.1570 15141377 PMC7167720

[85] Lu R Zhao X Li J Niu P Yang B Wu H. Genomic characterisation and epidemiology of 2019 novel coronavirus: implications for virus origins and receptor binding. Lancet. (2020) 395:565–74. 10.1016/S0140-6736(20)30251-8 32007145 PMC7159086

[86] Chen J Jiang Q Xia X Liu K Yu Z Tao W. Individual variation of the SARS-CoV-2 receptor ACE2 gene expression and regulation. Aging Cell. (2020) 19:e13168. 10.1111/acel.13168 32558150 PMC7323071

[87] Simões e Silva AC Silveira KD Ferreira AJ Teixeira MM. ACE2, angiotensin-(1-7) and Mas receptor axis in inflammation and fibrosis. Br J Pharmacol. (2013) 169:477–92. 10.1111/bph.12159 23488800 PMC3682698

[88] Felsenstein S Herbert JA McNamara PS Hedrich CM. COVID-19: immunology and treatment options. Clin Immunol Orlando Fla. (2020) 215:108448. 10.1016/j.clim.2020.108448 32353634 PMC7185015

[89] Gao X Zhou H Wu C Xiao Y Ren L Paranhos-Baccalà G. Antibody against nucleocapsid protein predicts susceptibility to human coronavirus infection. J Infect. (2015) 71:599–602. 10.1016/j.jinf.2015.07.002 26165609 PMC7127429

[90] Che X-Y Qiu L-W Liao Z-Y Wang Y Wen K Pan Y-X. Antigenic cross-reactivity between severe acute respiratory syndrome-associated coronavirus and human coronaviruses 229E and OC43. J Infect Dis. (2005) 191:2033–7. 10.1086/430355 15897988 PMC7109809

[91] Chen KYH Cheung M Burgner DP Curtis N. Toxic shock syndrome in Australian children. Arch Dis Child. (2016) 101:736–40. 10.1136/archdischild-2015-310121 27117838

[92] Streptococcal Toxic Shock Syndrome | 2010 Case Definition. Available online at: https://wwwn.cdc.gov/nndss/conditions/streptococcal-toxic-shock-syndrome/case-definition/2010/ (accessed August 31, 2020).

[93] Toxic Shock Syndrome (Other Than Streptococcal) | 2011 Case Definition. Available online at: https://wwwn.cdc.gov/nndss/conditions/toxic-shock-syndrome-other-than-streptococcal/case-definition/2011/ (accessed August 31, 2020).

[94] Cook A Janse S Watson JR Erdem G. Manifestations of toxic shock syndrome in children, Columbus, Ohio, USA, 2010–20171. Emerg Infect Dis. (2020) 26:1077–83. 10.3201/eid2606.190783 32442091 PMC7258457

[95] Krakauer T. Staphylococcal superantigens: pyrogenic toxins induce toxic shock. Toxins. (2019) 11:178. 10.3390/toxins11030178 30909619 PMC6468478

[96] Cheng MH Zhang S Porritt RA Arditi M Bahar I An insertion unique to SARS-CoV-2 exhibits superantigenic character strengthened by recent mutations. bioRxiv [Preprint]. (2020). 10.1101/2020.05.21.109272

[97] Beukelman T Xie F Chen L Baddley JW Delzell E Grijalva CG. Rates of hospitalized bacterial infection associated with juvenile idiopathic arthritis and its treatment. Arthritis Rheum. (2012) 64:2773–80. 10.1002/art.34458 22569881 PMC3409300

[98] Horneff G. Paediatric rheumatic disease: biologic therapy and risk of infection in children with JIA. Nat Rev Rheumatol. (2012) 8:504–5. 10.1038/nrrheum.2012.114 22801983

[99] Ibrahim A Ahmed M Conway R Carey JJ. Risk of infection with methotrexate therapy in inflammatory diseases: a systematic review and meta-analysis. J Clin Med. (2018) 8:15. 10.3390/jcm8010015 30583473 PMC6352130

[100] Nimmrich S Horneff G. Incidence of herpes zoster infections in juvenile idiopathic arthritis patients. Rheumatol Int. (2015) 35:465–70. 10.1007/s00296-014-3197-6 25583050

[101] Emmi G Bettiol A Mattioli I Silvestri E Di Scala G Urban ML. SARS-CoV-2 infection among patients with systemic autoimmune diseases. Autoimmun Rev. (2020) 19:102575. 10.1016/j.autrev.2020.102575 32376395 PMC7200134

[102] Fredi M Cavazzana I Moschetti L Andreoli L Franceschini F Airò P. COVID-19 in patients with rheumatic diseases in northern Italy: a single-centre observational and case–control study. Lancet Rheumatol. (2020) 2:e549–56. 10.1016/S2665-9913(20)30169-7 32838307 PMC7302769

[103] Haslak F Yildiz M Adrovic A Sahin S Koker O Aliyeva A. Management of childhood-onset autoinflammatory diseases during the COVID-19 pandemic. Rheumatol Int. (2020) 40:1423–31. 10.1007/s00296-020-04645-x 32661928 PMC7355083

[104] Götzinger F Santiago-García B Noguera-Julián A Lanaspa M Lancella L Carducci FIC. COVID-19 in children and adolescents in Europe: a multinational, multicentre cohort study. Lancet Child Adolesc Health. (2020) 4:653–61. 10.1016/S2352-4642(20)30177-2 32593339 PMC7316447

