# Supplementary material for: Pediatric Inflammatory Multisystem Syndrome and Rheumatic Diseases During SARS-CoV-2 Pandemic
Source: Front Pediatr. 2020 Dec 4;8:605807. doi: 10.3389/fped.2020.605807 (PMC7746854; doi:10.3389/fped.2020.605807)
Supplement: Supplementary file 1 [file Data_Sheet_1.docx]

Appendix 1: Different proposals for PIMS/MIS-C classification according to different public health committees from Europe, North America and WHO.

| **French Euro surveillance** (15) | **World Health Organisation** (40) | **UK Royal College of Paediatrics and Child Health** (41) | **US Center for Disease Control and Prevention** (42) |
| --- | --- | --- | --- |
| Children under 18 years of age with fever  AND  At least one of the following MAS, myocarditis, seritis Kawasaki-Like features (if evidence for SARS-CoV2 positive) or at least two items if pending missing virological results  Biologcial status :  Positive for current or recent SARS-CoV2 infection by RT-PCR, serology (confirmed PIMS) or COVID-19 exposure or CT scan evovative (Probable PIMS)  Pending or not performed in Possible PIMS (at least two criteria)  Excluded if  Negative for both SARS-CoV2 related PCR and serology | Children and adolescents 0–19 years of age with fever > 3 days    **AND**  Two of the following:  a) Rash or bilateral non-purulent conjunctivitis or muco-cutaneous inflammation signs (oral, hands or feet).  b) Hypotension or shock.  c) Features of myocardial dysfunction, pericarditis, valvulitis, or coronary abnormalities (including ECHO findings or elevated Troponin/NT-proBNP),  d) Evidence of coagulopathy (by PT, PTT, elevated d-Dimers).  e) Acute gastrointestinal problems (diarrhoea, vomiting, or abdominal pain).  **AND**  Elevated markers of inflammation such as ESR, C-reactive protein, or procalcitonin.  **AND**  No other obvious microbial cause of inflammation, including bacterial sepsis, staphylococcal or streptococcal shock syndromes.  **AND**  Evidence of COVID-19 **(**RT-PCR, antigen test or serology positive), or likely contact with patients with COVID-19. | 1. A child presenting with persistent fever, inflammation (neutrophilia, elevated CRP and lymphopaenia) and evidence of single or multi-organ dysfunction (shock, cardiac, respiratory, renal, gastrointestinal, or neurological disorder) with additional features (see listed in Appendix 2). This may include children fulfilling full or partial criteria for Kawasaki disease. 2. Exclusion of any other microbial cause, including bacterial sepsis, staphylococcal or streptococcal shock syndromes, infections associated with myocarditis such as enterovirus (waiting for results of these investigations should not delay seeking expert advice). 3. SARS-CoV-2 PCR testing may be positive or negative | - An individual aged <21 years presenting with fever*, laboratory evidence of inflammation**, and evidence of clinically severe illness requiring hospitalization, with multisystem (>2) organ involvement (cardiac, renal, respiratory, hematologic, gastrointestinal, dermatologic or neurological)   AND   - No alternative plausible diagnoses   AND   - Positive for current or recent SARS-CoV-2 infection by RT-PCR, serology, or antigen test; or exposure to a suspected or confirmed COVID-19 case within the 4 weeks prior to the onset of symptoms.   *Fever >38.0°C for ≥24 hours, or report of subjective fever lasting ≥24 hours **Including, but not limited to, one or more of the following: an elevated C-reactive protein (CRP), erythrocyte sedimentation rate (ESR), fibrinogen, procalcitonin, d-dimer, ferritin, lactic acid dehydrogenase (LDH), or interleukin 6 (IL-6), elevated neutrophils, reduced lymphocytes and low albumin  Additional comments:   - Some individuals may fulfil full or partial criteria for Kawasaki disease but should be reported if they meet the case definition for MIS-C. - Consider MIS-C in any pediatric death with evidence of SARS-CoV-2 infection. |

Appendix 2 :

**Royal College of Paediatrics and Child Health,** Clinical and laboratory features of PIMS:

| **Clinical** | | | **Laboratory** | | **Imaging and electrocardiogram (ECG)** |
| --- | --- | --- | --- | --- | --- |
| **All** | **Most** | **Some** | **All** | **Some** |  |
| 1. Persistent fever >38.5°C | Oxygen requirement | Abdominal pain | Abnormal Fibrinogen | Acute kidney injury | Echo and ECG – myocarditis, valvulitis, pericardial effusion, coronary artery dilatation |
|  | Hypotension | Confusion | Absence of potential causative organisms (other than SARS-CoV-2) | Anemia | Chest X ray – patchy symmetrical infiltrates, pleural effusion |
|  |  | Conjunctivitis | High CRP | Coagulopathy | Abdominal ultrasound – colitis, ileitis, lymphadenopathy, ascites, hepatosplenomegaly |
|  |  | Cough | High D-Dimers | High IL-10 (if available) * | CT chest – as for Chest X Ray – may demonstrate coronary artery abnormalities if with contrast |
|  |  | Diarrhea | High ferritin | High IL-6 (if available) * |  |
|  |  | Headache | Hypoalbuminaemia | Neutrophilia |  |
|  |  | Lymphadenopathy | Lymphopenia | Proteinuria |  |
|  |  | Mucus membrane changes | Neutrophilia in most – normal neutrophils in some | Raised CK |  |
|  |  | Neck swelling |  | Raised LDH |  |
|  |  | Rash |  | Raised triglycerides |  |
|  |  | Respiratory symptoms |  | Raised troponin |  |
|  |  | Sore throat |  | Thrombocytopenia |  |
|  |  | Swollen hands and feet |  | Transaminitis |  |
|  |  | Syncope |  |  |  |
|  |  | Vomiting |  |  |  |

*These assays are not widely available. CRP can be used as a surrogate marker for IL-6. CRP : C Reactive Protein , CK : Creatinine Kinase ; LDH : Lactate Dehydrogenase ; IL : InterLeukin
